# The Role of Oxidative Stress in the Relationship Between Periodontitis and Alzheimer’s Disease: A Review of the Literature

**DOI:** 10.3390/jpm15080384

**Published:** 2025-08-18

**Authors:** Konstantinos Antonios Papadakis, Aikaterini-El Doufexi, Mary S. Kalamaki, Evangelos Bourazanas, Evgenia Lymperaki

**Affiliations:** 1 Department of Biomedical Science, International Hellenic University, 57400 Thessaloniki, Greece; evlimper@ihu.gr; 2 Department of Preventive Dentistry, Periodontology and Dental Implant Biology, Dental School, Aristotle University of Thessaloniki, 54124 Thessaloniki, Greece; doufexi@dent.auth.gr; 3 Division of Technology and Sciences, American College of Thessaloniki, 55535 Thessaloniki, Greece; kalamaki@act.edu; 4 Chemistry School, Aristotle University of Thessaloniki, 54635 Thessaloniki, Greece; empouraza@chem.auth.gr

**Keywords:** periodontitis, Alzheimer’s disease, oxidative stress, neuroinflammation, reactive oxygen species, cognitive decline, mitochondrial dysfunction

## Abstract

Periodontitis, a chronic inflammatory disease affecting the supporting tissues of the teeth, has been linked to the onset of neurological diseases, including Alzheimer’s disease (AD). A primary mechanism connecting these two issues is oxidative stress caused by an imbalance between antioxidant defenses and reactive oxygen species (ROS) synthesis. This review compiles results from both animal and human studies that explore how oxidative stress resulting from periodontitis leads to neuroinflammation, mitochondrial dysfunction, and cognitive decline in AD. Studies in animals indicate that periodontal infections worsen brain oxidative damage, as evidenced by elevated lipid peroxidation markers, such as malondialdehyde (MDA), and indicators of oxidative DNA damage, including 8-hydroxy-2′-deoxyguanosine (8-OHdG). Additionally, significant reductions in crucial antioxidant enzymes, including superoxide dismutase (SOD) and glutathione peroxidase, along with neuroinflammation and cognitive deficits, are observed in mouse models of induced periodontitis. Supporting evidence from human studies reveals lower total antioxidant capacity (TAC) in individuals with both Alzheimer’s disease (AD) and periodontitis, as well as increased systemic oxidative stress markers, such as advanced oxidation protein products (AOPRs). These findings suggest a mechanistic relationship through oxidative stress pathways between periodontal inflammation and neurodegeneration. Given the extensive impact of periodontitis, enhancing periodontal health could be a viable strategy to reduce oxidative damage and lower the risk of cognitive decline. Further research is needed to clarify causality and to investigate antioxidant treatments aimed at preventing or slowing the progression of AD in patients with periodontal disease.

## 1. Introduction

Periodontitis is a chronic inflammatory disease affecting the teeth’s supporting structures—cementum, gingiva, the periodontal ligament, and alveolar bone. Its primary cause is an imbalance between microbial dysbiosis within the subgingival biofilm and the human immune response with significant effects on oral health and overall well-being, periodontitis is a leading cause of tooth loss worldwide and is characterized by the gradual degradation of periodontal tissues.

Periodontitis results from a complex interaction between bacterial infections, the host’s immune responses, and environmental factors such as smoking, diabetes, and poor oral hygiene. Major periodontal pathogens like *Porphyromonas gingivalis*, *Treponema denticola*, and *Tannerella forsythia* cause tissue damage by inducing chronic inflammation and oxidative stress. This continuous inflammatory reaction leads to alveolar bone resorption, collagen breakdown, and periodontal attachment loss [1].

Beyond its oral manifestations, periodontitis has been linked to systemic diseases such as cardiovascular disease, diabetes mellitus, respiratory infections, and neurological disorders, including Alzheimer’s disease [2,3]. Further emphasizing the importance of early detection and intervention, emerging data suggest that periodontitis-induced systemic inflammation and oxidative stress may be potential etiological factors for these disorders [4].

Given its high prevalence and possible systemic ramifications, periodontitis remains a significant public health concern. Modern strategies for controlling inflammation and restoring periodontal health include mechanical debridement, antibacterial treatments, and host modulation [3,5]. Ongoing research aims to improve diagnostic techniques and treatment approaches, enhancing clinical outcomes and reducing the broader health impacts associated with periodontal disease.

Affecting millions of people worldwide, Alzheimer’s disease (AD) is a progressive neurological illness and the most common cause of dementia [6]. It is characterized by cognitive decline, memory loss, and behavioral changes, which significantly impact patients’ quality of life [7,8]. Consequently, caregivers and healthcare systems experience substantial burdens. The pathophysiology of AD involves the accumulation of amyloid-beta (Aβ) plaques and hyperphosphorylation of tau protein, leading to neurofibrillary tangles, synaptic dysfunction, and chronic neuroinflammation. Additionally, factors such as oxidative stress, mitochondrial dysfunction, and genetic predispositions, including mutations in the apolipoprotein E (APOE) gene, further influence the progression of the disease [9]. 

Alzheimer’s disease (AD) is multifactorial and results from a combination of environmental and genetic factors. It can be classified as sporadic or familial (FAD) [10]. Approximately 5% of AD cases are familial, which arise from mutations in the APP, presenilin 1, or presenilin-2 genes, leading to the production of aggregating-friendly Aβ peptides. Apolipoprotein E4 (APOE4) expression, which transports lipids, cholesterol, and other hydrophobic substances into the brain [9,11], may be associated with sporadic variants. Additionally, environmental factors, such as psychological stress, are highly relevant to the etiology of sporadic AD and can also increase the risk of neurodegenerative diseases. Furthermore, genetic risk factors can exacerbate AD. The significance of these immune cells is emphasized by alterations or polymorphisms in numerous genes expressed by microglia and other myeloid cells [12,13,14]. While perivascular macrophages and monocytes from bone marrow can migrate to the central nervous system (CNS), especially during brain injury or systemic inflammation, microglia remain the predominant immune cell population within the CNS [13].

With chronic inflammatory diseases, including periodontitis, linked as possible risk factors for AD, emerging studies indicate a strong relationship between systemic inflammation and neurodegeneration [15,16]. The bidirectional link between neuroinflammation and peripheral inflammatory illnesses emphasizes the need to look at changeable risk factors, such as oral health, in the framework of cognitive decline and dementia avoidance. Understanding the fundamental causes and possible preventive measures is still a vital field of study since rising life expectancy causes the worldwide prevalence of AD to keep growing [17].

## 2. Scope and Literature Selection

This review is narrative in nature and aims to synthesize evidence from both animal and human studies investigating the role of oxidative stress in the relationship between periodontitis and Alzheimer’s disease (AD). The literature was sourced from PubMed, Scopus, and Google Scholar using the following keywords in various combinations: “periodontitis”, “Alzheimer’s disease”, “oxidative stress”, “Porphyromonas gingivalis”, “reactive oxygen species”, and “neuroinflammation”. Preference was given to peer-reviewed publications and recent systematic reviews from 2000 to 2025. Both preclinical and clinical studies were included to provide a comprehensive overview. As this is not a systematic review, no formal inclusion/exclusion protocol or PRISMA flowchart was used.

## 3. Overview of Alzheimer’s Disease and Its Systemic Context

AD is ranked seventh globally in 2019 among all causes of death. Alzheimer’s disease (AD) and certain types of dementia clearly have a significant impact on public health and life expectancy [18]. As the frequency of AD notably rises with age, the demographic most affected by the condition primarily consists of women over 65 [18]. Beginning with minor, often undetectable changes in the brain and progressively leading to noticeable cognitive impairments such as memory loss, difficulties with executive function, and language deficits, AD is characterized as a chronic, progressive neurodegenerative disorder where neurons sustain irreversible damage. Patients’ autonomy and quality of life are significantly impacted by communication barriers, confusion, and behavioral issues that accompany advancing neural degeneration [19,20]. In the later stages of the disease, individuals may lose the ability to perform basic tasks, including speaking, swallowing, and walking, which increases their dependence on caregivers and medical treatments. Early-onset Alzheimer’s disease (ELAD) and late-onset Alzheimer’s disease (LOAD) [21,22] define two distinct subgroups of AD based on the age of onset. The classification is established at 65 years; ELAD occurs before this threshold, while LOAD manifests later in life. The less common ELAD is typically associated with inherited genetic mutations in genes such as SORL1 (sortilin-related receptor) and TREM2 (triggering receptor expressed on myeloid cells), which are involved in amyloid processing and neuroinflammatory responses [23,24,25].

Although the pathogenesis of Alzheimer’s disease (AD) is mostly caused by hereditary inclination, some modifiable risk factors greatly influence the course of the disease. These elements comprise physical inactivity, tobacco use, poor dietary habits, limited educational attainment, hypertension, traumatic brain injury, type II diabetes, and cerebrovascular diseases, all of which are linked with systemic inflammation and vascular dysfunction and so aggravate neurodegenerative process [11].

The strong association between AD and other systemic disorders emphasizes the need for a multidisciplinary strategy for the control and avoidance of diseases. Given the common risk factors for metabolic and cardiovascular illnesses, public health campaigns have to provide lifestyle changes and early intervention as a top priority in order to lower AD and postpone its start priority. Cognitive health promotion also has great importance.

## 4. Epidemiological Evidence Linking Periodontitis and AD

Numerous epidemiological studies involving large population-based cohorts, retrospective analyses, and robust statistical methodologies have confirmed the statistical link between chronic periodontitis (CP) and Alzheimer’s disease (AD). A retrospective cohort study that used data from the Korean National Health Insurance Service applied Cox proportional hazards regression models to adjust for possible confounders, including smoking, alcohol use, and physical activity [26]. This analysis revealed a modest yet significant increase in the risk of AD among individuals afflicted with CP (AHR = 1.05, 95% CI = 1.00–1.11). A matched-cohort study utilizing Taiwan’s National Health Insurance Research Database demonstrated that individuals with a decade-long exposure to CP had a 1.707-fold increased risk of developing AD (95% CI = 1.152–2.528, *p* = 0.0077), indicating a strong statistical association [26]. Further research has revealed similar patterns; specifically, individuals with untreated chronic periodontitis exhibited an increased risk of Alzheimer’s disease relative to those who received periodontal treatment (AHR = 1.14, 95% CI = 1.04–1.24), thereby substantiating the classification of periodontal disease as a modifiable risk factor. A review pooling longitudinal data concluded that chronic periodontitis (CP) increases the risk of Alzheimer’s disease (AD) by approximately 1.5 to 2 times, depending on the severity and duration of the disease [27]. Although these studies suggest a positive association, the effect sizes remain modest, and residual confounding continues to be a concern, particularly given the complex interplay between systemic inflammation, genetic predisposition, and lifestyle factors in the pathogenesis of AD. Future research employing Mendelian randomization, machine learning, and randomized controlled trials related to periodontal interventions may offer stronger causal inferences and further validate this association. Numerous epidemiological studies utilizing large population-based cohorts, retrospective analyses, and rigorous statistical methodologies have supported the statistical link between chronic periodontitis (CP) and Alzheimer’s disease (AD) (Table 1).

## 5. Mechanisms Underlying the Association

Several biological mechanisms have been proposed to explain the link between periodontitis and AD:

### 5.1. Neuroinflammation and Systemic Inflammatory Mediators

The progression of periodontal disease contributes to localized and systemic inflammation through multiple biological mechanisms. A primary pathway involves the release of bacterial endotoxins, such as lipopolysaccharides (LPSs), which trigger an exaggerated immune response from the host, resulting in tissue destruction and persistent inflammation [1]. Furthermore, dysbiotic oral microbiota stimulates the hyperactivity of polymorphonuclear leukocytes (PMNs), leading to increased oxidative stress and excessive production of reactive oxygen species (ROSs), which further exacerbates periodontal tissue destruction [29]. In addition to local tissue damage, these inflammatory processes contribute to systemic inflammation by elevating the circulating levels of key pro-inflammatory cytokines, including tumor necrosis factor-alpha (TNF-α), transforming growth factor-beta (TGF-β), and interleukin-1 (IL-1) [27]. These cytokines perpetuate the destruction of periodontal tissue and contribute to the systemic inflammatory burden, thereby influencing disease processes in distant organ systems.

Periodontitis, acting as a chronic inflammatory stimulus, is associated with various systemic conditions. Strong epidemiological and mechanistic evidence indicates a link between periodontitis and diabetes mellitus, as chronic inflammation promotes insulin resistance and disrupts glycemic control [30]. Likewise, periodontitis has been associated with cardiovascular diseases; periodontal inflammation likely plays a role in atherogenesis by causing endothelial dysfunction, systemic oxidative stress, and heightened arterial plaque buildup [2,31]. Additionally, periodontitis has been associated with adverse pregnancy outcomes, including preterm birth and low birth weight, likely due to systemic inflammatory mediators affecting placental function [32]. Respiratory conditions such as chronic obstructive pulmonary disease (COPD) have also been linked to periodontal disease, as aspiration of periodontal pathogens and inflammatory byproducts may exacerbate pulmonary inflammation [33].

More recently, research has investigated the potential role of periodontal disease in neurodegenerative disorders, particularly Alzheimer’s disease (AD). Chronic systemic inflammation, driven by conditions such as periodontitis, has been proposed to contribute to neuroinflammation and neurodegeneration [27]. Periodontal pathogens, including Porphyromonas gingivalis, have been identified in the brains of AD patients, suggesting a possible connection to amyloid-beta accumulation and neurofibrillary tangle formation, both hallmark features of AD pathology. Furthermore, systemic inflammatory cytokines arising from periodontal inflammation may compromise the blood–brain barrier, permitting inflammatory mediators and bacterial byproducts to enter the central nervous system and worsen neurodegeneration [34].

### 5.2. Direct Bacterial Invasion into the Brain

Recent evidence suggests that Porphyromonas gingivalis (*P. gingivalis*), a major periodontal pathogen, plays a role in Alzheimer’s disease (AD) pathogenesis. Studies have detected *P. gingivalis* DNA and virulence factors in the brains of AD patients [35]. Gingipains, cysteine proteases secreted by *P. gingivalis*, degrade tight junction proteins in the blood–brain barrier (BBB), facilitating bacterial translocation into the central nervous system (CNS). This breakdown of the BBB increases neuroinflammation and accelerates neurodegeneration Chronic periodontal inflammation elevates systemic levels of pro-inflammatory cytokines (e.g., TNF-α, IL-1β) and matrix metalloproteinases (e.g., MMP-9), which contribute to blood–brain barrier disruption by weakening tight junctions and altering endothelial function [36]. This compromised barrier allows bacterial components such as lipopolysaccharide (LPS) and gingipains to infiltrate the brain parenchyma, triggering microglial activation and sustained neuroinflammation. Additionally, periodontal pathogen-induced endotoxemia has been associated with increased BBB permeability in animal models, further supporting the concept of an oral–systemic–neurodegenerative axis [37].

**Figure 1:**
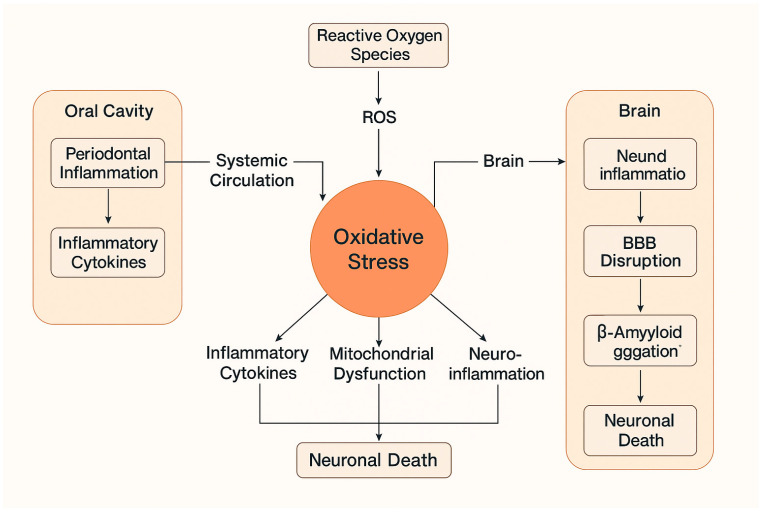
Link between periodontal disease and the role of oxidative stress on the initiation and progression of Alzheimer’s disease.

Lipopolysaccharides (LPSs) from *P. gingivalis* have been identified in AD brains, triggering microglial activation and an exaggerated inflammatory response Microglial activation leads to increased production of pro-inflammatory cytokines such as TNF-α and IL-1β, contributing to neuronal damage. Chronic exposure to *P. gingivalis* and its byproducts may promote amyloid-beta (Aβ) aggregation, a hallmark of AD pathology. Gingipains have been shown to enhance Aβ production and impair clearance mechanisms, further exacerbating neurotoxicity (Figure 1).

The antimicrobial hypothesis of AD suggests that Aβ may act as an innate immune response to microbial invasion. Chronic infection with *P. gingivalis* may trigger persistent Aβ deposition, leading to plaque formation and cognitive decline Studies in animal models demonstrate that chronic oral infection with *P. gingivalis* results in neuroinflammation, hippocampal damage, and memory impairment [1].

Therapeutic strategies targeting *P. gingivalis* have shown promise in preclinical models. Small-molecule inhibitors of gingipains reduce bacterial load, neuroinflammation, and Aβ deposition, highlighting the potential for periodontal-targeted interventions in AD management (36)Maintaining oral health through periodontal therapy may help reduce systemic inflammation and its impact on cognitive decline [34]. The evidence linking *P. gingivalis* to AD underscores the importance of oral health in neurodegenerative disease prevention.

### 5.3. Oxidative Stress: Biological Basis and Relevance

Oxidative stress is a biological condition marked by an imbalance between the production of reactive oxygen species (ROSs) and the body’s capacity to detoxify these reactive intermediates or repair the damage they inflict [38]. Reactive oxygen species (ROSs), including free radicals and peroxides, are natural byproducts of cellular metabolism, especially during mitochondrial energy production. While low to moderate levels of ROSs play essential roles in cell signaling and immune defense, excessive ROSs can damage cellular structures such as proteins, lipids, and DNA, leading to cellular dysfunction and apoptosis.

The primary sources of oxidative stress include endogenous metabolic processes, such as mitochondrial respiration and enzymatic reactions involving nicotinamide adenine dinucleotide phosphate (NADPH) oxidase, as well as exogenous factors like environmental pollutants, ultraviolet (UV) radiation, and toxins [4]. Over time, the buildup of oxidative damage contributes to aging and the development of various diseases, including cancer, cardiovascular conditions, neurodegenerative disorders, and metabolic syndromes [39].

Mitochondrial dysfunction is both a source and consequence of oxidative stress, contributing to a self-perpetuating cycle of cellular injury. In both periodontitis and Alzheimer’s disease, excessive ROSs production impairs mitochondrial respiratory complexes and damages mitochondrial DNA (mtDNA), further amplifying redox imbalance and promoting apoptosis in periodontal and neuronal cells [40].

Numerous studies have linked oxidative stress to the development of chronic diseases. In neurodegenerative disorders like Alzheimer’s and Parkinson’s diseases, oxidative stress leads to neuronal damage by inducing mitochondrial dysfunction, increasing neuroinflammation, and promoting protein aggregation [41].

The human body has evolved several antioxidant defense systems to counteract oxidative stress. These include enzymatic antioxidants, such as superoxide dismutase (SOD), catalase, and glutathione peroxidase, as well as non-enzymatic antioxidants, such as vitamin C, vitamin E, and glutathione Dietary intake of antioxidants from fruits, vegetables, and other sources has been shown to mitigate oxidative damage and improve overall health outcomes [42].

Oxidative stress plays a critical role in cellular homeostasis and disease progression. Understanding the mechanisms of oxidative stress and enhancing antioxidant defenses through lifestyle and pharmacological interventions may help prevent or mitigate chronic diseases. Future research should focus on targeted therapies to regulate oxidative stress and its pathological consequences.

## 6. The Relationship Between Saliva and the Brain

There is a bidirectional relationship between saliva and the human, which is mediated by variable pathways: the neural pathway, with direct anatomic routes along several cranial nerves; the intranasal pathway; the lymphatic pathway; the sublingual route; and the transport of bacteria and inflammatory cytokines to the brain via the peripheral bloodstream. Furthermore, the oral–gut–brain axis via the vagal nerve must be considered [37].

In the hippocampus, oral *Treponema denticola* and *Porphyromonas gingivalis* are identified as inducers of inflammation and beta-amyloid (Aβ) accumulation [43]. *Treponema pallidum* was identified in the axons of peripheral nerves [43,44]. Further anatomical neural pathways include the facial nerve via the nervus intermedius, with its sensory fibers for the anterior tongue and soft palate, and the chorda tympani, the parasympathetic innervation to the submandibular and sublingual salivary glands, which induces salivary secretion. The glossopharyngeal nerve—the parasympathetic innervation of the parotid gland—is another anatomic connection to the brain [43]. The glossopharyngeal nerve has a unique role as a neural pathway for immune-to-brain communication [44].

Transient bacteremia happens frequently in everyday life during tooth brushing, flossing, and chewing [45], allowing oral bacteria to migrate to other tissues and organs and also enter the brain. *Porphyromonas gingivalis* can even actively enter endothelial cells [46]. The bacteria-induced, pro-inflammatory cytokines result in systemic low-grade chronic inflammation, can induce neuroinflammation, and influence brain function.

The gut–brain axis is a bidirectional pathway linking the gut with the brain via the brainstem [47].

The precondition for the last two pathways is a leaky blood–brain barrier (BBB). Systemic inflammatory mediators in the bloodstream can induce a leaky BBB, which allows substances from the bloodstream to enter the brain across the BBB. This triggers a vicious cycle of neuroinflammation, BBB permeability issues, and cognitive decline, which is common in neurodegenerative disorders such as AD [47,48].

Recent evidence supports the use of salivary biomarkers as a non-invasive diagnostic bridge between oral health and neurodegenerative disease. Oxidative stress markers such as malondialdehyde (MDA), 8-hydroxy-2′-deoxyguanosine (8-OHdG), and total antioxidant capacity (TAC) have been detected at elevated levels in the saliva of individuals with periodontitis and cognitive impairment. These biomarkers reflect systemic redox imbalance and correlate with both periodontal inflammation and AD severity. The ease of sample collection and patient compliance make saliva an attractive fluid for early screening, especially in elderly or cognitively vulnerable populations Integrating salivary diagnostics into routine dental and geriatric care could offer a novel, low-cost strategy for identifying individuals at risk for neurodegenerative progression.

## 7. Periodontitis-Induced Oxidative Stress

Affecting the supporting components of the teeth, periodontitis is a chronic inflammatory disease causing increasing tissue deterioration and, finally, tooth loss. Oxidative stress, which comes from an imbalance between reactive oxygen species (ROS) generation and the body’s antioxidant defense system, is one of the main pathogenic processes behind periodontitis [49]. In aggravating periodontal inflammation, tissue damage, and disease progression, oxidative stress is absolutely vital.

The pathogenesis of periodontitis is driven by the host’s immunological response to bacterial biofilms, which recruits neutrophils, macrophages, and other immune cells. As part of their defense against periodontal infections, these cells produce high levels of ROS, including superoxide anions, hydrogen peroxide, and hydroxyl radicals [1]. On the other hand, too-high ROS generation can harm periodontal tissues, including DNA damage, lipid peroxidation, and extracellular matrix protein degradation.

Furthermore, aggravating inflammatory responses, oxidative stress [50], kappa B (NF-κB) and mitogen-activated protein kinase (MAPK), contributing to mitochondrial dysfunction and inflammation (1). This self-perpetuating cycle of oxidative stress and inflammation hastens periodontal deterioration and systemically occurring problems [51].

Several indicators have been found to evaluate periodontitis oxidative stress levels. Among these are 8-hydroxydeoxyguanosine (8-OHdG), a sign of oxidative DNA damage, and malondialdehyde (MDA), a marker of lipid peroxidation [52,53]. Periodontitis patients generally have lower antioxidant enzyme activity, including catalase, glutathione peroxidase, and superoxide dismutase (SOD), which further supports the part oxidative stress plays in periodontal pathogenesis [54,55].

In periodontitis, oxidative stress has systemic effects rather than limited consequences within the mouth cavity. Rising ROS levels and systemic inflammation help to explain the development of many chronic diseases, including diabetes mellitus, cardiovascular disease, and neurodegenerative disorders, including Alzheimer’s disease. The systematic spread of oxidative stress indicators and inflammatory cytokines emphasizes the interdependence of periodontal and general health.

### 7.1. Studies in Animals

Many studies on animal models have examined the association between periodontitis-induced oxidative stress and pathology akin to Alzheimer’s disease (AD). With an eye toward key pathways like oxidative stress, neuroinflammation, and amyloid-beta (Aβ) pathology, these studies use mouse models to investigate how periodontitis fuels neurodegeneration. The findings suggest that ongoing periodontal infection results in systemic oxidative damage, mitochondrial dysfunction, and cognitive abnormalities on par with those observed in AD patients. Table 1 presents a general summary of these animal experiments coupled with the numerous methods of inducing periodontitis and their neurodegenerative consequences (Table 2).

In hippocampus tissues of rats with ligature-induced periodontitis, studies have shown notable increases in oxidative stress markers like malondialdehyde (MDA) and 8-hydroxy-2′-deoxyguanosine (8-OHdG), therefore indicating increased oxidative DNA damage and lipid peroxidation [59]. These oxidative changes cause degeneration and malfunction of neurons. Furthermore, contributing to neuronal injury is LPS from Porphyromonas gingivalis, which activates microglia via the NOX4 pathway, therefore producing higher reactive oxygen species (ROSs), interleukin-6 (IL-6), and interleukin-8 (IL-8).

Within the hippocampal tissue, chronic exposure to *Porphyromonas gingivalis* triggers neuroinflammation, evidenced by increased levels of pro-inflammatory cytokines, including tumor necrosis factor-alpha (TNF-α), IL-1β, and IL-6. *P. gingivalis*-LPS exacerbates Toll-like receptor 4 (TLR4) signaling, further intensifying neuronal inflammation and impairing cognitive processes and memory retention in animal models [57]. Furthermore, chronic periodontal infection is associated with elevated amyloid-beta (Aβ) accumulation and tau phosphorylation, reflecting the neuropathological features of Alzheimer’s disease.

Cognitive abnormalities, including poor memory recall and spatial learning, consistently manifest in behavioral tests conducted on animal models of periodontitis. These behavioral deficits align with scientific findings related to oxidative stress, inflammation, and amyloid pathology.

These animal studies taken together clearly suggest that periodontitis-induced oxidative stress and neuroinflammation are fundamental causes of Alzheimer’s-like neurodegeneration. Future studies should investigate remedies, including antioxidant therapy or periodontal treatments, to reduce the negative impact of periodontitis on cognitive function. Elevated oxidative stress markers and decreased antioxidant defenses in individuals with both conditions indicate that managing periodontal health may be essential in alleviating systemic oxidative stress and potentially slowing cognitive decline. Further longitudinal and interventional studies are necessary to clarify causal relationships and explore therapeutic strategies targeting oxidative stress in this context.

### 7.2. Studies in Humans

While animal models provide valuable insights into the mechanisms linking periodontitis with Alzheimer’s disease (AD), human studies are crucial for understanding the clinical relevance of these findings. Evidence from human research indicates that individuals with both Alzheimer’s disease and periodontitis exhibit heightened oxidative stress markers and diminished antioxidant defenses.

Several cross-sectional and cohort studies have highlighted a correlation between chronic periodontitis and increased systemic oxidative stress in humans. For instance, individuals with periodontitis have been observed to have elevated levels of advanced oxidation protein products (AOPPs) and reduced total antioxidant capacity (TAC), suggesting a systemic oxidative imbalance.

Furthermore, human studies have demonstrated an association between periodontal disease severity and cognitive decline. Research involving large population-based cohorts has revealed that untreated chronic periodontitis is associated with an increased risk of developing Alzheimer’s disease. This correlation underscores the potential impact of periodontal health on cognitive function.

Interventional studies in humans have also examined the effects of periodontal treatment on systemic inflammation and oxidative stress. Findings suggest that improving periodontal health can lead to reductions in systemic inflammatory markers and oxidative stress levels, potentially mitigating the risk of neurodegeneration.

Despite these promising findings, further longitudinal and interventional studies are needed to clarify the causal pathways and to explore the potential benefits of periodontal therapy in slowing or preventing cognitive decline associated with Alzheimer’s disease [32,40,60].

## 8. Discussion

The major clinical effects of the interplay between oxidative stress resulting from Alzheimer’s disease (AD) and periodontal disease (PD) demand a coordinated approach to therapy. Given the increasing number of studies linking periodontal health to oxidative stress and systemic inflammation, addressing periodontal health may help reduce neurodegeneration and cognitive decline.

One of the main clinical results of this relationship is the need for a multidisciplinary approach combining dental and neurological treatment. Periodontal disease should be considered as a potential AD-modifying element even though it is often ignored in comprehensive health control. Routine periodontal screenings for elderly people—especially those at risk for cognitive decline—should be standard practice in dental and medical settings. Similarly, individuals diagnosed with AD or moderate cognitive impairment (MCI) should get regular periodontal examinations to lower the probable effects on neurodegeneration and systemic inflammatory load [34].

Additionally, essential components of clinical treatment include early diagnosis and biomarker development. Since periodontitis causes systemic inflammation and oxidative stress, its presence may signal the onset of neurodegenerative processes. Utilizing salivary and blood biomarkers, such as pro-inflammatory cytokines (e.g., tumor necrosis factor-alpha [TNF-α], interleukin-1β [IL-1β], IL-6), oxidative stress markers (e.g., malondialdehyde, 8-hydroxydeoxyguanosine), and periodontal pathogen DNA, could aid in the early identification of individuals at higher risk for Alzheimer’s disease (AD). Future research should primarily focus on validating and incorporating these biomarkers into routine screening protocols [39].

Controlling periodontal disease as a preventive tool against cognitive decline is one area of growing interest. Evidence suggests that periodontal therapy—including mechanical debridement, antimicrobial treatments, and improved oral hygiene practices—may reduce systemic inflammation and oxidative stress, thereby potentially slowing neurodegeneration. Moreover, it is important to instruct AD patients’ caregivers on maintaining oral hygiene since individuals with cognitive decline usually have declining self-care abilities, which leads to worse periodontal health [61]. Furthermore, research into viable treatments to lower inflammation and oxidative stress could be antibacterial and probiotic therapy targeted at the oral microbiota.

The involvement that oxidative stress plays in AD and periodontal disease suggests that anti-inflammatory and antioxidant therapies could have twofold benefits. Many studies have investigated how efficiently antioxidants such as resveratrol, vitamin E, curcumin, and glutathione precursors reduce oxidative damage. Comparably, anti-inflammatory medications, including TNF-α inhibitors and IL-6 blockers, have been examined for their prospective neuroprotective effects. Moreover, polyphenols and statins demonstrate efficacy in reducing systemic inflammation; consequently, more research in the settings of periodontal and neurological disorders would be justified [62].

Public health initiatives stressing the link between dental and cognitive health ought to be included in public life. Regarding the need for dental hygiene in reducing systemic inflammation and its possible influence on brain health, primary care physicians, neurologists, dentists, and other healthcare providers should counsel patients—especially older persons and those with metabolic or cardiovascular comorbidities—about this. One reasonably cheap approach to help reduce the load of neurodegenerative diseases is to include periodontal assessments in routine medical visits.

The key focus of further research should be longitudinal studies aiming at proving causality between AD and periodontal disease. Clear causal relationships are still elusive, even if present data points demonstrate a strong correlation. Long-term observational studies tracking dental health and cognitive performance over decades are crucial to understand whether periodontal disease directly affects AD pathogenesis or is an early indication of systemic inflammation.

Moreover, interventional clinical studies are needed to assess whether treating periodontal disease could reduce systemic inflammation and improve cognitive function. Studies evaluating the effects of mechanical debridement, antimicrobial treatments, and host-modulating strategies on neuroinflammation biomarkers and cognitive performance could offer an interesting study of the possible advantages of oral health interventions for AD avoidance.

Moreover, advances in precision medicine and customized treatment strategies will help us to better understand the common pathophysiology linking periodontal disease to AD. The identification of individuals most likely to have both diseases, made achievable by genetic and molecular profiles, could enable focused preventative and treatment strategies. Furthermore, research on the microbiome could motivate tailored probiotic or microbiome-modulating treatments designed to reduce pathogenic bacterial burdens and accompanying inflammation [63].

More investigation of shared pathophysiological mechanisms, including oxidative stress, mitochondrial dysfunction, and neuroinflammation, is needed. Advanced imaging technology and biomolecular investigations could assist in clarifying how periodontal infections and inflammatory mediators transit the blood–brain barrier and lead to dementia. Understanding these routes could lead to new therapeutic targets and treatments concurrently treating periodontal and neurodegenerative disease processes [22,64].

A shared biological background between periodontitis and Alzheimer’s disease may also lie in the concept of immunosenescence and inflammaging—chronic, low-grade inflammation that increases with age. Aging impairs the resolution of inflammation and skews immune responses, leading to heightened production of ROSs and pro-inflammatory cytokines such as IL-6 and TNF-α. This dysfunctional immune state may exacerbate periodontal tissue breakdown and promote neuroinflammation, potentially accelerating the onset or progression of AD. Exploring the interactions between oxidative stress, senescent immune cells, and the aging microenvironment could provide deeper insights into common age-driven mechanisms of both diseases [39,65].

At last, the growing evidence linking periodontal disease, oxidative stress, and AD underlines the need for integrated healthcare approaches stressing oral health as part of a more general attempt to lower neurological risk. The key targets of the next research should be validation of biomarkers, long-term and interventional studies, and evaluation of focused therapy techniques. By addressing the oral–systemic link, healthcare providers can progress towards early intervention, better patient outcomes, and possible strategies to halt the progression of Alzheimer’s disease.

This review is narrative in nature and does not follow a formal systematic methodology, which may introduce selection bias in the choice of literature. While we have prioritized peer-reviewed and representative studies, the absence of standardized inclusion criteria limits the reproducibility of this review. Furthermore, oxidative stress biomarkers vary significantly in sensitivity, specificity, and sampling method across studies, complicating direct comparisons. The majority of available data are derived from cross-sectional or animal studies, limiting causal inferences. Longitudinal clinical trials and mechanistic human studies are needed to validate the proposed pathways linking periodontitis, oxidative stress, and neurodegeneration.

## 9. Conclusions

Recently, periodontal disease has been associated with numerous systemic diseases. One of them is Alzheimer’s disease. The common pathophysiological mechanism is probably the increased systemic inflammation, including oxidative stress. It is well established that periodontal disease and periopathogens generate an environment with high oxidative stress. Its products can deteriorate not only periodontal disease and the oral cavity, but can also affect brain function through systemic inflammation.

## 10. Future Directions

Retention of teeth has been associated with higher cognitive longevity in the elderly. Dementia, AD, and type II diabetes can all be associated with periodontal disease. Moreover, the polymicrobial biofilm of PD and the immune response to inflammation that can be associated with apolipoprotein gene allele 4 (APOE e4), a susceptibility gene to both lipid metabolism and resulting dyslipidemia, and also predisposition to infection, can be a strong genetic association between PD and AD [22].

Case–control clinical studies in patients with PD and AD and interventional studies with treatment of PD disease and its effect on the progression of AD, and the role of oxidative stress are needed to clarify the exact pathway of the biological link between the two diseases. A growing body of evidence suggests that oxidative stress can induce epigenetic alterations, including DNA methylation, histone oxidation, and microRNA dysregulation, which may contribute to sustained inflammation and neurodegeneration. In both periodontitis and Alzheimer’s disease, ROS-mediated epigenetic changes have been implicated in the silencing of antioxidant genes and the activation of pro-inflammatory pathways. These modifications may explain how transient exposures to periodontal pathogens or oxidative bursts have lasting biological consequences. Future studies investigating oxidative epigenetics could help identify stable biomarkers of risk and uncover novel therapeutic targets that address inflammation and neurodegeneration at the gene-regulation level [25].

## Figures and Tables

**Table 1:** Representative epidemiological studies investigating the association between periodontitis and Alzheimer’s disease.

Study	Association	Mechansism	Result
[26]	Statistical		Link between AD and PD
[27]	Risk for developing AD	Matched-cohort	Patients with PD have a 1.7-fold increased risk of developing AD
[28]	Review	Review of the literature	Overall relative risk for patients with PD to develop AD is 1.5–2

**Table 2:** Summary of animal studies linking periodontitis-induced oxidative stress to neurodegeneration.

Study	Model	Periodontitis Induction Method	Key Findings
[56]	Rats	Ligature-induced periodontitis	-Increased hippocampal TNFa, IL-1β levels and oxidative stress marker 8-OHdG. -Limited apoptotic changes and neurodegenerative signs.
[37]	Mice	Oral infection with Pophyromonas gingivals	-Increase hippocampal TNFa, IL-1β, IL-6 expression. -Neurodegenaration, microgliosis, astrogliosis, and extracellular amyloid-beta (Aβ) accumulation. -Elevated tau phospholylation and cognitive impairment.
[57]	Mice	Administration of Porphyromonas gingivalis LPS	-Impaired spatial learning, memory, and cognitive functions. -Activated microglia and astrocytes in cortex and hippocampus. -Increased TNF- a, IL-1β, IL-6, and IL-8 expression via TLR4 signalling pathway activation.
[58]	Human Microglia Cells	Porphyromonas gingivalis LPS stimulation	-NOX4-mediated reactive oxygen species (ROS) production and increased IL-6 and IL-8 secretion. -Reduced neuronal viability and elevated phosphorylated tau levels. -Enhanced neuroinflammatory response, potentially contributing to Alzheimer’s pathology.

## Data Availability

No new data were created or analyzed in this study. Data sharing is not applicable to this article.
